# Antibiotic use for upper respiratory tract infections in children: A cross-sectional survey of knowledge, attitudes, and practices (KAP) of parents in Greece

**DOI:** 10.1186/1471-2431-11-60

**Published:** 2011-07-05

**Authors:** Sotiria G Panagakou, Νikos Spyridis, Vassiliki Papaevangelou, Kalliopi M Theodoridou, Georgia P Goutziana, Maria N Theodoridou, George A Syrogiannopoulos, Christos S Hadjichristodoulou

**Affiliations:** 1 Department of Hygiene and Epidemiology, Faculty of Medicine, University of Thessaly, Larisa, Greece; 2 Second Department of Paediatrics, Aglaia Kyriakou Children's Hospital, University of Athens, Athens, Greece; 3 Department of Epidemiology, Medical Statistics, Athens School of Public Health, Athens, Greece; 4 First Department of Paediatrics, Agia Sofia Children's Hospital, University of Athens, Athens, Greece; 5 Department of Pediatrics, University of Thessaly, Faculty of Medicine, General University Hospital, Larisa, Greece

## Abstract

**Background:**

Upper respiratory tract infections (URTIs) are common in children. The cause of URTIs is usually viral, but parents' attitudes often contribute to inappropriate prescription of antibiotics, promoting antibiotic resistance. The objective of this study was to document and analyse parental beliefs on antibiotic use for children with URTIs in Greece, a country with high levels of antibiotic use and antibiotic resistance.

**Methods:**

A knowledge-attitude-practice questionnaire was developed and distributed to Greek parents caring for children who were 5-6 years old, between January and July of the same school year. The sample of the study contained parents from all geographic areas of Greece.

**Results:**

The majority of Greek parents (80%) believed that UTRIs are mostly self-limited, although 74% of them expected to receive antibiotics when such a diagnosis was given. Earache was the most common reason for which parents expected antibiotics (45%). Greek parents rarely gave antibiotics to their children without medical advice (10%) and most (88%) believed that unnecessary antibiotic use drives antibiotic resistance and they were happy to receive symptomatic therapy if instructed by their physician. Almost 70% of parents confused antibiotics with other medicines used for symptomatic therapy for a child with URTI.

**Conclusion:**

Greek parents have a trusted relationship with their paediatrician and rarely give antibiotics without medical advice, indicating that parents contribute less than expected to antibiotic misuse. Parents also appreciate the benign course of most URTIs and the fact that unnecessary antibiotic use is harmful. More time needs to be invested in educating mostly physicians on the potential benefit from reducing antibiotic prescribing for children with URTI.

## Background

Over the past decade, the emergence of antibiotic resistance has been recognised as an important public health problem because discovery of new antibiotics is no longer keeping pace with the spread of highly resistant bacterial pathogens [1]. Prescription of antibiotics for upper respiratory tract infections (URTIs) is very common practice in paediatrics [2-6], although there is sufficient evidence to support the viral origin of most of those illnesses [7,8]. However, even some of the bacterial illnesses (such as otitis media and sinusitis) are usually self-limited, and antibiotic treatment is unnecessary. A recent report has documented that even if common RTIs are caused by bacteria, the probability of their resolution without the administration of antibiotics is high [9]. Unnecessary prescription of antibiotics is the main driver for the development of antibiotic resistance [10-12] and both paediatricians [7,13] and parents contribute to this problem [2,3,12].

According to the latest data from the European Centre for Disease Prevention and Control, Greece has the highest rate of antibiotic consumption amongst 27 European member states [14]. There is currently no clear evidence to explain the reasons leading to antibiotic overuse. To determine these contributing factors in Greece, we designed a knowledge, attitude and practice (KAP) questionnaire for parents in relation to antibiotic use for common URTIs in a national, descriptive, cross-sectional study.

## Methods

The KAP study measures the knowledge (what people know), attitude (how they feel) and practices (how they behave) of the population under study.

Our study sample contained parents of all geographical areas of Greece (Peloponnesus, islands, Athens, Northern Greece, and Central Greece). A school-based stratified geographical clustering sampling was used to select a representative sample of students, whose parents were asked to fill in the questionnaire by a letter explaining the importance of the subject (i.e., the definition and nature of URTIs and the problem of injudicious antibiotic use) and their cooperation in the study. The population of children aged 5 or 6 years old in Greece was approximately 240,000, according to the census of 2001. The number of required geographical clusters was calculated to be 200 based on the mean number of students in each class (n = 40). In total, 100 kindergarten (age 5 years) and 100 elementary schools (first year students, aged 6 years) were randomly selected and stratified according to the population of children in each region. The sample size was estimated taking into consideration the geographical cluster sampling methodology (double the needed sample) and the expected percentage of non-responders (20%). Approval was given by the Ministry of Education to contact the parents through the selected students. A total of 7704 parents with children aged between 5 and 6 years old were invited to participate in the survey. The survey was conducted between January and July of the same school year. The study design and questionnaire was approved by the educational institute of the Ministry of Education and General Assembly of the Medical Faculty at the University of Thessaly (reference number: 401/15 - 02 - 06).

The scientific team, which constructed the questionnaire, included one epidemiologist, two paediatric specialists on infectious diseases, one statistician and one MD researcher. The main objective of the study was to include questions based on the methodology of KAP studies conducted in other countries, but to adapt them to the Greek situation and culture. To accomplish this goal, manuscripts and published papers describing similar research and methodological issues were studied. Initially, the draft of the questionnaire included 77 questions. To assure the clarity, accuracy and consistency of the questions, the questionnaire was pre-tested among 30 parents. The scientific team evaluated the pre-test results and after excluding and modifying some questions, the final questionnaire was designed (see additional file 1), containing 50 questions. Half of the questions originated from the scientific team (questions 4, 8, 12, 13, 14, 17, 19, 23, 24, 25, 26, 28, 30, 31, 34, 35, 36, 37, 38, 40, 43, 44, 45, 48 and 50), while the rest of the questions were copied from other similar questionnaires of published papers retrieved from the Internet.

The KAP questionnaire, except for the demographic data (questions 1-14), was structured into three main sections: (1) knowledge that parents possess to understand issues concerning URIs and antibiotics (section A, questions 15-23); (2) attitude concerning parental feelings, beliefs or conceptions towards antibiotic use in URIs (section B, questions 24-38); and (3) practise concerning the ways that parents demonstrate their knowledge and attitude through their actions (section C, questions 39-50). Most questions were based on the 5-point Likert scale, which expresses emotions: strongly disagree - disagree - uncertain - agree - strongly agree; frequency: always - most of the time - often - sometimes - never; and quantity: very much - plenty - not much - a little - none. Chronic disease was defined as recurrent URTIs such as asthma.

A more detailed description of all methodological aspects of the study, including the design, development and reliability of the questionnaire, and sampling procedures have been presented in a previous paper [15]. The current study aimed to describe the parents' KAP profile without any association of the answers with demographics to identify possible risk factors for antibiotic misuse.

### Data analysis

The data were entered into a database using the Epi Info program and were analysed by SPSS version 15.0. Descriptive analysis was conducted by using frequencies of the variables and the 95% confidence intervals (CIs) were calculated. Questionnaires that were poorly completed (< 50% of the questions) were excluded from the statistical analysis [15].

## Results

We collected 5312 questionnaires out of 7704 that were initially disseminated, representing a response rate of 68.95%. Forty-eight questionnaires were excluded later because of poor completion. The highest response rate was from Northern Greece (74.36%), while the lowest was found in the islands (62.27%). The demographic characteristics of the respondents are demonstrated in Table 1. We found that the vast majority of the respondents (78.5%) were mothers and 70% of parents characterised their household income as moderate. No more than 2% of parents mentioned that they were uninsured, while nearly 10% declared immigrant status. Approximately half of the parents had finished high school. Only 5.7% of parents reported a single family status.

**Table 1:** Parents' demographic characteristics (questions 1-14)

Demographic characteristics	Number of respondents	Per cent
Female	4168	78.5%
Median age	36.2 years old (IQR = 33 - 40)	
Mean number of children	2.1 children	
Insured	5204	98%
Access to health care system*	4402	82.9%
High family income*	780	14.7%
Moderate family income*	3710	69.8%
Low family income*	564	10.6%
School graduates	2903	54.6%
College/University graduates	2133	40.2%
Urban residents	3209	60.4%
Immigrants	513	9.7%
Single parents	301	5.7%
Child with chronic diseases (i.e. asthma)	792	14.9%

*Self - assessment as perceived by the parents at the time of the survey

### Knowledge

Ninety percent (CI: 89%-90.7%) of parents obtained information on judicious antibiotic use from paediatricians. Thirty-seven percent (CI: 34.2%-39.3%) of parents obtained information from the media, while just 2.2% (CI: 1.8%-2.6%) declared that they "had never been informed" about appropriate antibiotic use (question 15).

When parents were given a list of drugs including antibiotics, antipyretics, bronchodilators and expectorants, and were asked to distinguish antibiotic products from other drugs, 30.4% of them made no mistakes at all, 42.1% made one mistake and 27.5% made two or more mistakes (question 16).

**Figure 1:**
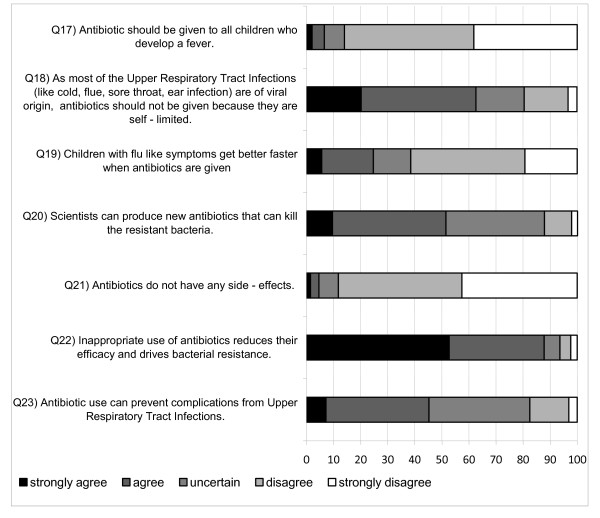
**Parents' responses (%) to questions related to knowledge (Q17 to Q23)**.

Figure 1 demonstrates the responses to questions 17-23 that were present in the knowledge section. A total of 88% (CI: 85.2%-90.5%) of the parents were aware of the fact that antibiotic misuse drives bacterial resistance, but 24.7% (CI: 23.1%- 26.5%) would still give antibiotics because they thought that recovery would be quicker. Finally, half (CI: 49.4%-53.7%) of the respondents believed that new stronger antibiotics are always available.

### Attitude

**Figure 2:**
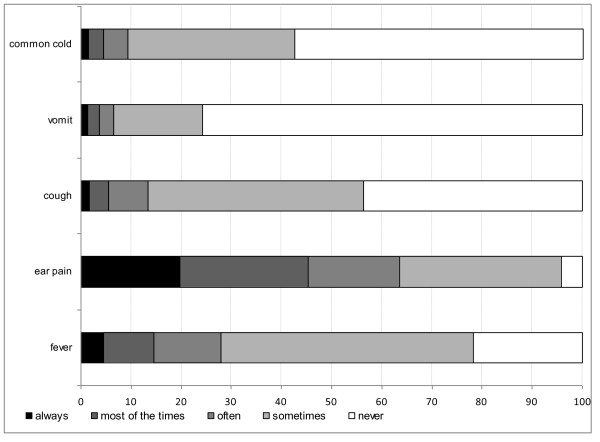
**Parental expectations to receive antibiotics in relation to various symptoms**.

Once a child develops URTI symptoms (question 24), over 40% of parents would seek a paediatric opinion within 2-3 days (mean duration, 2.38 days; SD = 0.959). When parents were asked for possible treatment options, they chose antibiotics as a possible therapy 74% of the time. In addition, when parents were asked for the most common symptoms leading to a paediatric visit (including cough, fever, runny nose, ear ache, sore throat, hoarseness and change of behaviour) (question 26), 95.4% of symptoms were a runny nose (CI: 94.8%-95.9%), often accompanied by other symptoms. Figure 2 indicates parental expectations for antibiotic use in relation to URTI symptoms (question 27). Ear ache was the most common symptom for which parents expected to receive antibiotics [45,4% (CI: 43.1%-47.8%)], while symptoms of the common cold seldom led to a similar expectation [4.5% (CI: 3.8%-5.3%)].

Ten percent of parents (CI: 7.5%-14.2%) would consider giving their children antibiotics without previous medical advice (question 28). More precisely, a lack of money or time would lead to over the counter antibiotic consumption in just 1.2% (CI: 0.7%-1.8%) of the participants, while 2.2% (CI: 1.6%-3%) would administer antibiotics to their child because they thought that symptoms were not important enough to visit the paediatrician.

**Figure 3:**
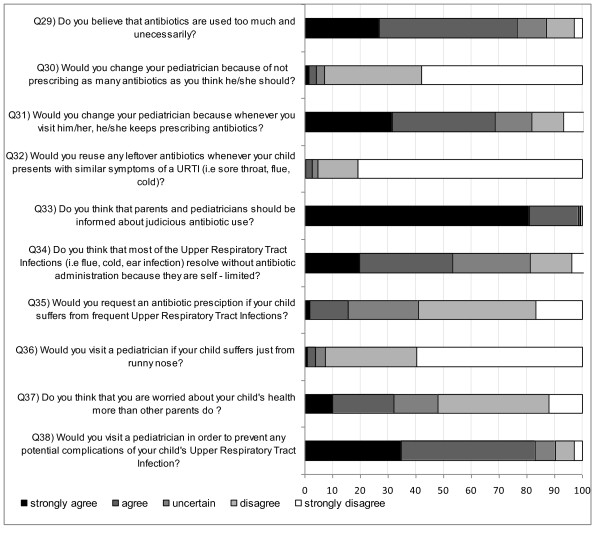
**Parents' responses (%) to questions related to attitude (Q29 to Q38)**.

Figure 3 illustrates the answers for questions 29-38. Seventy-eight percent (CI: 74%-79.1%) of parents believed that antibiotics are used too much. Nevertheless, 7% (CI: 17.3%-20.5%) would change paediatricians if he/she did not prescribe antibiotics often enough. Similar results were concluded from question 42 as 13.4% (CI: 12.5%-14.4%) of parents stated that they would be dissatisfied if the paediatrician did not give an antibiotic prescription for URTI symptoms. Ninety-eight percent (CI: 96.4%-100.6%) believed that further information should be given to both parents and paediatricians regarding judicious antibiotic use. Finally, 20% (CI: 18.4%-21.5%) of the responders believed that URTIs are not self-limited.

### Practice

**Figure 4:**
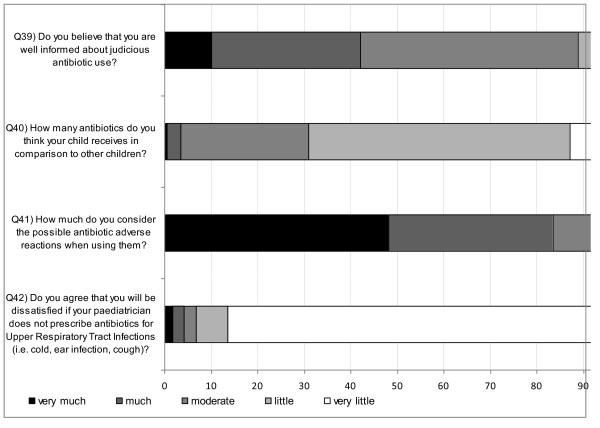
**Parents' responses (%) to questions related to practice (Q39 to Q42)**.

**Figure 5:**
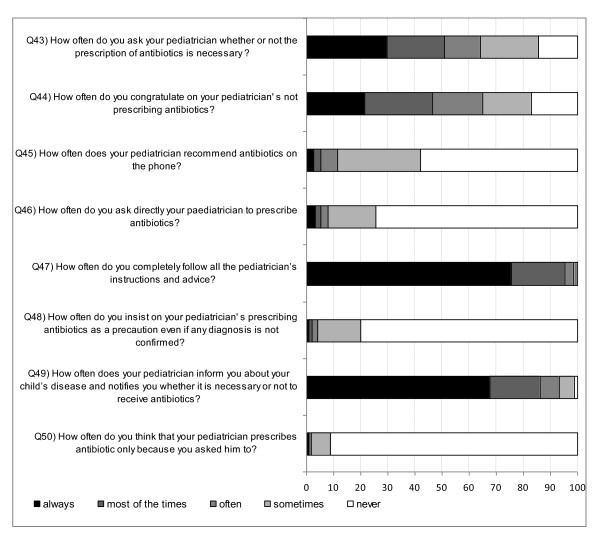
**Parents' responses (%) to questions related to practice (Q43 to Q50)**.

Figure 4 and Figure 5 illustrate the answers to questions 39-50. More than 2/3 of the parents declared that paediatricians (CI: 66.2%-68.8%) provided sufficient information regarding diagnosis and therapy, while 14.5% (CI: 13.5%-15.4%) of parents never questioned the paediatricians if antibiotic administration was necessary. Forty-two percent (CI: 40.8%-43.5%) of parents received antibiotic recommendation from their paediatrician over the phone. A total of 25% (CI: 23.2%-25.6%) of parents did not always follow the paediatrician's advice.

## Discussion

This is the first published population-based study on KAP of parents from Greece in relation to treatment of URTIs. The overall response rate was almost 69%, which is satisfactory, although it varied amongst different regions of Greece. The highest rate of a low educational status was noted in the islands, indicating that this could be a factor for the low response rate of the islands. On the other hand, in Northern Greece, since the immigrant population was less compared with other regions, it is likely that the respondents understood the Greek language and medical terms better than in the other regions, and therefore, a higher proportion participated in the study. In contrast, the islands have the highest percentage of immigrants (data shown in a previous paper) [15].

According to the geographical clustering methodology, we had to double the sample required, and therefore, this required a large sample size. The number of clusters was also increased to cover the likelihood of non-respondents (20%). Finally, we wanted to achieve the highest accuracy and consistency during the elaboration of responses and make safe conclusions about regional comparisons.

This study showed that Greek parents and physicians have a trusted relationship because most parents were happy with the information provided to them and they would not change their private paediatrician if antibiotics were used too much or too little.

Parents also believed that URTIs are mostly self-limited (80%) although 74% of them expected to possibly receive antibiotics when such a diagnosis was given. However, it is incorrect to assume that 74% of the parents desired only antibiotic therapy because the majority of them also preferred other drugs given for symptomatic therapy. In contrast, the answers of question 27 (Figure 2) can be considered as more accurate. A runny nose accompanied by other URTI symptoms was the most common reason for a paediatric visit (95%), while ear ache was the most common diagnosis for which parents would expect to receive antibiotics (45%). Greek parents rarely gave antibiotics to their children without consulting their paediatrician (10%) and 88% of them appreciated that unnecessary antibiotic use drives antibiotic resistance. Interestingly, almost 70% of parents confused antibiotics with other medicines used for symptomatic relief for a child with URTI symptoms.

Limitations of the study were associated with a poor recall of an URTI experience and antibiotic use. Therefore, parents' knowledge, attitude and practices may not always be consistent with their actual behaviour. Invalid answers may also have occurred because of embarrassment. Additionally, the language used to form the questions may not have been fully understood by parents of low socioeconomic status (because of the use of medical terms) or immigrants (because of the language barrier) leading to inaccurate answers or even to no answers at all. It was also difficult to document if the responses were in fact a reflection of the paediatricians' view on antibiotic use and not the parents' view. Finally, subjective appreciation of URTI symptoms (cough, runny nose, and ear ache) may have influenced the responses.

This is not the first study indicating public misconceptions with regard to antibiotic use for common URTIs. In our study, although parents believed that most URTIs are self-limiting, they expected to receive antibiotics when this diagnosis was made. Similarly, in a web-based questionnaire among a sample of the general Dutch population, Cals et al showed that nearly half of the responders (47%) incorrectly identified antibiotics as being effective in treating viral infections [16]. In the same study, the term "acute bronchitis" raised an immediate expectation for an antibiotic prescription similar to "ear ache-otitis", as shown in our data.

Greek parents also reported that information regarding unnecessary antibiotic use and resistance came from their private paediatricians, which is different from findings of a public survey published by Hawkings et al where respondents reported that most information regarding antibiotic resistance was derived from the media [17]. In the same study, patients had a low sense of personal ability to help contain this problem, while our data showed that parents were willing to assist by reducing antibiotic use if suggested by their physician. These different responses between studies are possibly a reflection of the difference in health care systems. The great majority of Greek children have regular follow-up by private consultant paediatricians who are accessible either on the phone or with a home/office visit. This leads to a close and trusted relationship between the parent-child and the physician. In the big islands, there are paediatricians practicing in the hospitals or in the health centres, or even practicing as private paediatricians. In the smaller islands, only general physicians are available to examine children.

Amongst European countries, Greece has the highest antibiotic consumption rates [14]. Antibiotic consumption in Greece is approximately 33 DDD/1000 inhabitants per day, while the median rate is not greater than 19 DDD/1000 inhabitants per day. Additionally, a recent European Commission report indicated that Greece also has the highest over the counter antibiotic sales amongst 27 EU countries [18]. In Greece, parents have free access to all types of antibiotics despite a specific legislation forbidding antibiotic use without a prescription. In a recent survey that took place in the capital of Greece, Athens, volunteers presented to pharmacies asking for ciprofloxacin and co-amoxiclav to document if it is possible to obtain antibiotics without a prescription [19]. Co-amoxiclav was given in 100% of cases while ciprofloxacin was given in 53%. The current study showed that over the counter use was very low but it is unclear how this reflects real life practice.

If all the above findings are representative for Greece, who is to "blame" for the fact that Greece has a high antibiotic consumption and resistance? There are two likely explanations: (1) physicians are overprescribers failing to follow guidelines or (2) physicians misinterpret parental expectations for antibiotic use.

### Are doctors overprescribers?

Currently, there is strong evidence to support a "no prescribing policy "for URTI and such guidelines have already been implemented in other European countries [20]. Recently published observational studies suggest that antibiotics show little benefit in preventing complications of mastoiditis following acute ototis media, quinsy following a sore throat and pneumonia following URTI/bronchitis [21]. However, physicians are reluctant to follow such guidelines and they fear that not prescribing antibiotics for URTIs can lead to treatment failure and significant side effects. Evidence from the United Kingdom indicates that regardless of current evidence and governmental pressure to minimise prescribing for self-limited viral infections, primary care physicians continue to prescribe antibiotics by miscoding the infections treated ('diagnostic drift') indicating that the raw guidelines are of no real benefit [22]. This also suggests that the key decision on whether to prescribe antibiotics depends on the child's clinical condition regardless if this is caused by a viral pathogen.

### Do doctors misinterpret parental expectations?

The specialty of general practitioners in Greece has progressed during the last few years, but there are still few general practitioners and they are mainly located in health centres of villages, small towns and small islands. Therefore, the vast majority of children are directly referred to paediatricians or to children's hospitals. Results from the current study indicated that Greek parents do not apply pressure to their paediatrician to prescribe antibiotics. Other studies have also shown that most parents seek consultation of a physician to determine whether antibiotics are needed [23,24] and patient satisfaction is not correlated with receipt of antibiotics but with time spent by the clinician [6]. Ethnic and cultural background might also be a significant factor affecting the rate of prescribing antibiotics [6], although in our study population this parameter was not examined. Contrary to the perception of many physicians, few parents will seek treatment from another paediatrician if antibiotics are not prescribed. Greek parents will rush to their paediatrician within 2 days of symptom onset, indicating a low threshold for consultation. If paediatricians perceive this attitude as a request to prescribe antibiotics, a cycle of expectation is created for subsequent illness, whereby when similar symptoms are experienced, the parent and child return expecting another prescription perceiving that the antibiotic, and not the natural course of the disease, resolved their child's symptoms. This cycle creates several concerns, which include unnecessary antibiotic use, circulation of resistant bacterial pathogens in the community and equally important high health care costs.

### Which are the future directions?

All studies that attempt to explore the reason for overprescribing and overuse of antibiotics have a single initiative: to find new tools in the fight against antibiotic resistance and consequently reduce morbidity, mortality and health care costs. There is sufficient evidence to support the notion that high prescribing rates of antibiotics drive antibiotic resistance [11,25] and primary care accounts for 80%-90% of all antibiotic prescriptions. In paediatrics, most of those prescriptions are given for uncomplicated viral infections of the upper respiratory tract [5]. Published data mostly reflect adult antibiotic use, and therefore, making safe conclusions about antibiotic consumption in children is limited. Greece is a high antibiotic consumer, which is important at a local and European level. When structuring an intervention policy, the results from this study might be a useful tool for other EU countries with similarly high levels of antibiotic prescribing. Parental beliefs, fears and expectations play an important role in consulting behaviour and determining whether an antibiotic is prescribed. Parents fear serious illness and worry that they will not be able to recognise the symptoms. This leads to frequent consultations for common URTIs, and subsequently, unnecessary antibiotic use. Interventions need to target different areas of this problem [26]. Parents should be given more information through a longer consultation time and interactive booklets [6]. Parents should also be encouraged to avoid seeing their doctor too early and toο frequently when their child develops symptoms of a common cold. Setting a realistic expectation about the likely duration of illness could reduce parental anxiety and rates of visits. Parents value a thorough examination, explanation, reassurance and advice or guidance more than a prescription for antibiotics [2]. Younger generations should also be focused on and taught from a very early age that medicines are not always the answer to self-limited medical problems, and URTIs should be used as a typical example. Over the counter antibiotic use is not acceptable practice and the Greek government has to act swiftly on this issue. The magnitude of the problem is not well documented and conflicting evidence makes this process difficult to understand. We consider that this issue is more severe in adults than in children, but in the resistance chain it makes no much difference. Finally, physicians' views on similar KAP surveys should be recorded. The current study showed that parents play a much lesser role than paediatricians on antibiotic overuse, indicating that determining what doctors practice for URTI treatment is probably the key to tackle this major issue. At the local and European level, a well structured intervention is required, which could be the result of similar studies in other countries with high prescribing habits.

## Conclusion

Greek parents have a trusted relationship with their paediatrician and rarely give antibiotics without medical advice, indicating that they contribute less than we expected on antibiotic misuse and over the counter antibiotic consumption. Parents also appreciate the benign course of most URTIs and the fact that unnecessary antibiotic use is harmful. We need to invest more time in educating mostly physicians on the potential benefit of reducing antibiotic prescribing for children with URTI.

## Competing interests

The authors declare that they have no competing interests.

## Authors' contributions

CSH, MNT, VP, GAS and SGP designed the study. SGP, GPG and KMT collected the data. CSH, SGP, GPG and KMT performed the statistical analysis and interpreted the results. CSH, NS and SGP wrote the manuscript. CSH, GAS, MNT VP and NS provided valuable insight for revising the manuscript. All authors read and approved the final manuscript.

## Pre-publication history

The pre-publication history for this paper can be accessed here:

http://www.biomedcentral.com/1471-2431/11/60/prepub

## Supplementary Material

Additional file 1 **Questionnaire**. Final form of the questionnaire disseminated to the parents Click here for file
